# Prognosis of extracolonic findings on clinical computed tomographic colonography: A single-center experience

**DOI:** 10.1371/journal.pone.0315601

**Published:** 2025-02-28

**Authors:** Hideto Tomimatsu, Keita Fujimoto, Taketo Suto, Masayuki Matsuo

**Affiliations:** 1 Radiology Department, Japanese Red Cross Gifu Hospital, Gifu City, Gifu Prefecture, Japan; 2 Radiology Department, Gifu University Graduate School of Medicine, Gifu City, Gifu Prefecture, Japan; Institute of Post Graduate Medical Education and Research, INDIA

## Abstract

Computed tomographic colonography (CTC) is crucial for colorectal cancer screening and secondary examinations, often revealing extracolonic findings with unclear significance. This study retrospectively evaluated the impact of such findings on the prognosis and management of 103 patients (mean observation period [OP]: 2183 days), categorized according to the CT Colonography Reporting and Data System (C-RADS). The distributions were 17% (mean OP: 2,821 days) for E1, 38% for E2 (2,759 days), 6% for E3 (2,150 days), and 39% for E4 (1,338 days). Eighteen patients were further examined, but no treatment-related changes were observed in the E2 or E3 cases. Six of nine E4 patients responded to treatment (mean OP: 1,286 days). Kaplan–Meier analysis revealed worse prognosis for E4 (mean survival: 10.1 years) than for E1–E3 combined (5.6 years) (*p* < 0.0001). E4 findings are key in guiding treatment. The concordance rate between the clinical (Past E) and revised (Revised E) categories was high (0.83, 95% confidence interval: 0.77–0.88). The prognoses differed significantly between Revised E1–E3 (mean survival: 10.0 years) and Revised E4 (6.2 years) (*p* < 0.0001). Although E4 significantly affects prognosis, E2 and E3 had limited effects on treatment, highlighting the need for further study to improve diagnostic accuracy.

## Background

Computed tomographic colonography (CTC) is less invasive than colonoscopy and barium enemas and has garnered significant attention in various large-scale clinical trials because of its potential utility in colorectal cancer screening and analysis [1–3]. CTC frequently determines extracolonic lesions, but the implications of these results for treatment plan modifications remain unclear. In the CT Colonography Reporting and Data System (C-RADS), these findings are categorized under the E category [4]. As a relatively new tool for colorectal investigation, CTC plays a growing role in clinical settings. In the European context, CTC is predominantly used for secondary assessments or follow-up examinations [5]. The United States Preventive Services Task Force, a key authority in preventive medicine, recommends grade-A colorectal screening for individuals aged 50–75 years every 5 years, with CTC considered a viable screening modality [6]. In Japan, CTC has been covered by insurance since 2012, but it is limited to cases with suspected colorectal malignancies. Furthermore, CTC is often used as an alternative screening tool for patients with positive fecal occult blood test results when colonoscopy is not feasible, providing an essential option for opportunistic screening and comprehensive colorectal assessment.

## Purpose

This study aimed to evaluate the clinical implications of extracolonic findings detected through CTC, specifically to determine the correlation between extracolonic lesions and prognosis in patients undergoing clinical CTC and to identify the association of such findings with their clinical trajectories.

## Materials and methods

### Patients

This study was approved by our institutional review board, and written informed consent was obtained from all participants prior to their inclusion. A total of 103 patients underwent clinical CTC examinations at Gifu University Hospital between May 2012 and February 2013. The cohort consisted of 59 males (mean age: 67.3 years; range: 40–85 years) and 44 females (mean age: 66.9 years; range: 42–88 years). Clinical indication–based CTC examinations, including follow-up for suspected colorectal abnormalities, were performed.

### CT imaging protocol

CTC was conducted following a complete colonoscopy using a 64-row multi-detector CT (750HD, GE Healthcare) without a tagging unit. Patients underwent plain or contrast-enhanced CT scans in prone or lateral and supine positions, with automatic exposure control set at a standard dose for preoperative assessments (supine and lateral noise index [NI] = 12 and supine NI = 10). An iodine dose of 600 mg/kg (Iomeron 350 mgI, Eisai Co., Ltd.) was administered over 30 s for contrast-enhanced CT. We used a bolus tracking method for the dynamic study. An automatic carbon dioxide insufflator (PROTOCO2L, EIDIA) was used to dilate the colon. Prone or lateral and supine images were acquired in a single breathhold. Image reconstruction used iterative reconstruction (ASiR 30%, GE Corporation) with a slice thickness of 1.25 mm and an interval of 1.25 mm without any gap for CTC.

### Image analysis

The CTC images were analyzed using an image workstation (Advantage workstation 4.2, GE Healthcare), and clinical reports were generated using diagnostic software (ShadeQuest, Yokokawa Medical). Four radiologists with varying levels of experience (one with 3 years, two with 4 years, and one with 14 years) participated in the readings. All readings were subsequently reviewed and confirmed by the most experienced radiologist, who had 14 years of experience and had conducted > 300 CTC examinations.

All reports were investigated to identify extracolonic findings and categorized according to their respective E categories outlined in the C-RADS. Lesion descriptions, including those of lymph nodes and similar findings, were classified according to suspicion level. Findings of “possible” or higher were categorized as E4. Findings with lower suspicion levels were classified as E3.

For the present study purpose, all CT images were re-evaluated to ensure greater accuracy in E-category classification. This review was independent of the E categories used in clinical practice. Additional re-evaluations were conducted between June and August 2024. Three gastrointestinal radiologists, with 6, 12, and 26 years of experience, respectively, reassessed the E categories for all extracolonic lesions to reach a consensus. For the sake of clarity throughout this paper, the clinically used E categories will be referred to as “Past E” and the re-evaluated ones will be referred to as “Revised E.”

### Statistical analysis and impact assessment

The patients’ electronic medical records were reviewed from the time of examination until treatment, follow-up, or death. The list of patients who provided consent was pseudonymized, ensuring linkable anonymity, and their prognoses and clinical outcomes were tracked through electronic medical records from December 29, 2023, to January 29, 2024.

Analyses were performed to assess the effect of identifying lesions categorized as Past E2 or higher on changes to the treatment plan. We evaluated additional diagnostic examinations triggered by extracolonic findings on CTC, such as CT, magnetic resonance imaging (MRI), positron emission tomography-CT (PET-CT), biopsy, and cytology. Electronic medical records were used to track all examinations to evaluate their influence on the treatment strategy.

We determined if complete surgical resection, any form of chemotherapy, or radiation therapy resulted in at least one instance of partial response or complete response. The treatment outcomes, specifically the ability to achieve a clinical response on the basis of extracolonic lesion identification, were confirmed by reviewing the patients’ clinical records and treatment histories.

## Results

**Fig 1:**
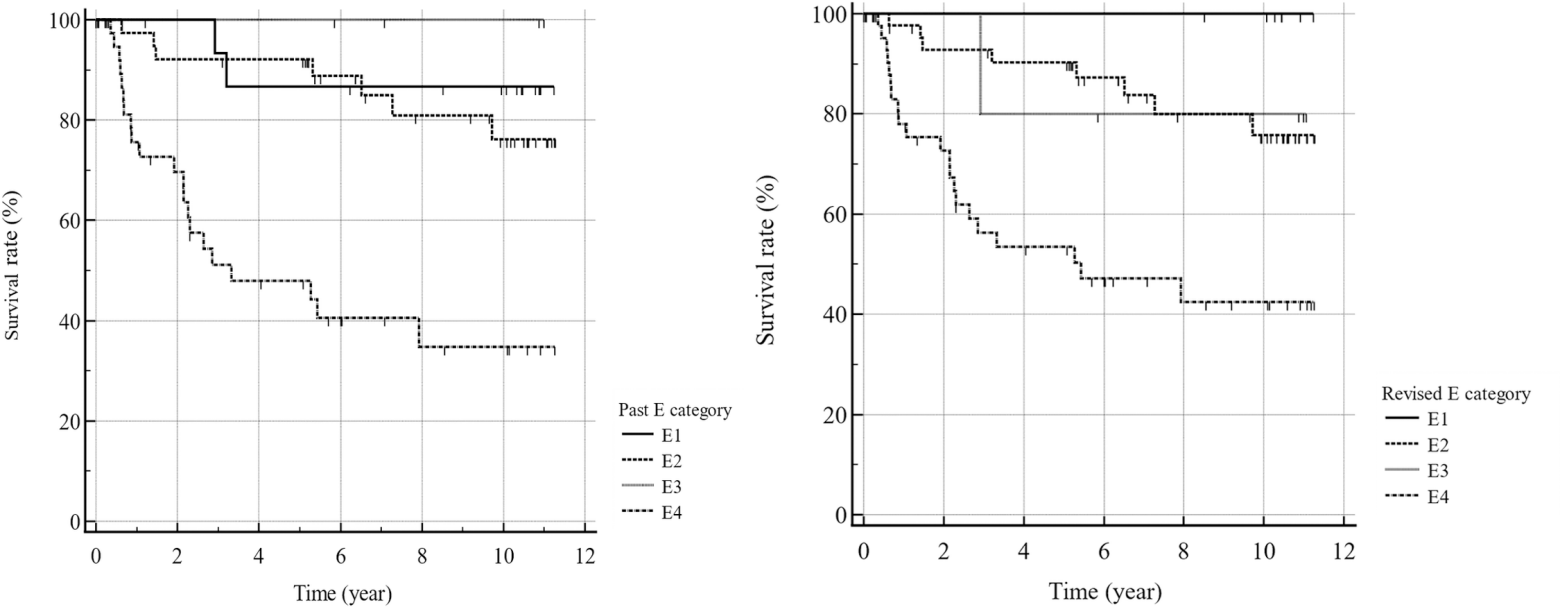
Kaplan–Meier survival curves for each Past (left) and Revised (right) E category. The survival curves for the Past E and Revised E categories show differences in overall trends between category E4 showed a poor prognosis and the trends of the E2 and E3 curves intersected. The 5-year survival rates were 87%, 92%, 100%, and 48% for the Past E1, E2, E3, and E4 (p < 0.0001, log-rank test) categories and 100%, 90%, 80%, and 53% for the Revised E1, E2, E3, and E4 categories, respectively (*p* < 0.0001, log-rank test). The survival curves showed differences in the E1 and E3 trends between the Revised E and Past E categories.

The primary indications for examination included colorectal tumor (58%), gastric tumor (18%), gynecological disease (6%), incomplete endoscopy (5%), and other reasons (13%). Nonionic transvenous iodine-based contrast agents were used in 61% of the cases. Among the 103 cases (mean observation period [OP]: 2,183 days), the distribution in the Past E category was E1, E2, E3, and E4 in 18 (17%, mean OP: 2,821 days), 39 (38%, 2,759 days), 6 (6%, 2,150 days), and 40 (39%, 1,338 days), respectively (Fig 1). Significantly, five E4 cases (5% of the total and 12.5% within E4) demonstrated incidental abnormalities unrelated to the primary disease, which had not been previously diagnosed. Among these, two cases (endometriosis with colonic adhesions and renal pelvic carcinoma) underwent surgical intervention and were pathologically confirmed (mean OP: 3,151 days). The remaining three cases (metastatic renal tumor, pelvic tumor, and abdominal aortic aneurysm) received no treatment because of disease progression (mean OP: 411 days). The distribution of patients in the Revised E category was 7 in E1 (7%, mean OP: 3,749 days), 44 in E2 (43%, mean OP: 2,633 days), 6 in E3 (6%, mean OP: 2,538 days), and 46 in E4 (45%, mean OP: 1,466 days) (Table 1).

**Table 1:** Distributions of patients in the past and revised E categories and mean observation period.

E Category	Number of Patients		Mean OP (days)	
	Past E category	Revised E category	Past E category	Revised E category
E1	18	7	2,821	3,749
E2	39	46	2,759	2,633
E3	6	6	2,150	2,538
E4	40	46	1,338	1,466

Observation period (OP) refers to the time elapsed between the initial diagnosis and either the patient’s death or the last confirmed survival date in the medical records.

In this study, the E4 category was observed more frequently in the general population. The mean OP was shorter for the E4 category than for the other categories.

The concordance rate between the Past E and Revised E categories was high at 0.83 (95% confidence interval [CI]: 0.77–0.88), reflecting some discrepancies (Table 2). In the cross-tabulation between the Past E and Revised E categories, a trend toward increasing categories was observed, with 16 cases showing an upward shift and five showing a downward shift due to re-evaluation. The most frequent change was from Past E1 to Revised E2, primarily due to the discovery of microcystic lesions and other minor findings. Additionally, six cases were reclassified from Past E1, E2, and E3 to Revised E4.

**Table 2:** Cross-tabulation for the past and revised E categories.

Category E	Revised E1	Revised E2	Revised E3	Revised E4
**Past E1**	6	9	2	1
**Past E2**	1	34	1	3
**Past E3**	1	3	3	2
**Past E4**	0	0	0	40

The most frequent change was from Past E1 to Revised E2, primarily due to the discovery of microcystic lesions and other minor findings. Additionally, six cases were reclassified from Past E1, E2, and E3 to Revised E4.

Moreover, 18 patients were referred for further examination due to Past E findings, including 4 with E2, 5 with E3, and 9 with E4 findings. These supplementary examinations included CT, MRI, PET-CT, cytology, and biopsy. No adverse events were associated with these examinations. Four patients were diagnosed with benign conditions in the case of E2 category findings. Meanwhile, five patients demonstrating Past E3 results underwent observational follow-up without any treatment intervention. None of the patients in the Past E2 or E3 categories required further examinations that necessitated changes to the treatment plan (mean OP for E2: 2,889 days; mean OP for E3: 2,976 days). Of the nine patients classified under the Past E4 category who were re-examined, six achieved a positive response to treatment, including complete surgical resection or partial response to chemotherapy (mean OP: 1,286 days). One of the nine patients who had an incidental discovery of an abdominal aortic aneurysm discovered was scheduled for treatment after management of the primary lesion but unfortunately died due to other causes before undergoing this treatment (OP: 162 days) (Table 3).

**Table 3:** Outcome and mean observation period (OP) for additional examinations for past E-category patients.

Past E Category	Outcome	Number of Patients	Mean OP (days)
E2	Benign lesion	4	2,880
E3	Observation, no treatment	5	2,976
E4	Positive response (resection/chemotherapy)	6	1,286
	*Recurrence/metastasis*	*4*	*695*
	*Worsening of concurrent malignancy*	*1*	*825*
	*Second malignancy*	*1*	*4,109*
	No response to chemotherapy	2	496
	Died before scheduled treatment	1	162

A positive response to treatment refers to a significant reduction in lesion size or complete resection of the lesion, either through surgical intervention or chemotherapy.

Recurrence/metastasis refers to the reappearance of the primary tumor or its spread to other organs during the observation period.

Scheduled for treatment but died refers to patients who had been diagnosed with conditions requiring treatment for abdominal aortic aneurysm but passed away before the planned treatment could be administered.

**Fig 2:**
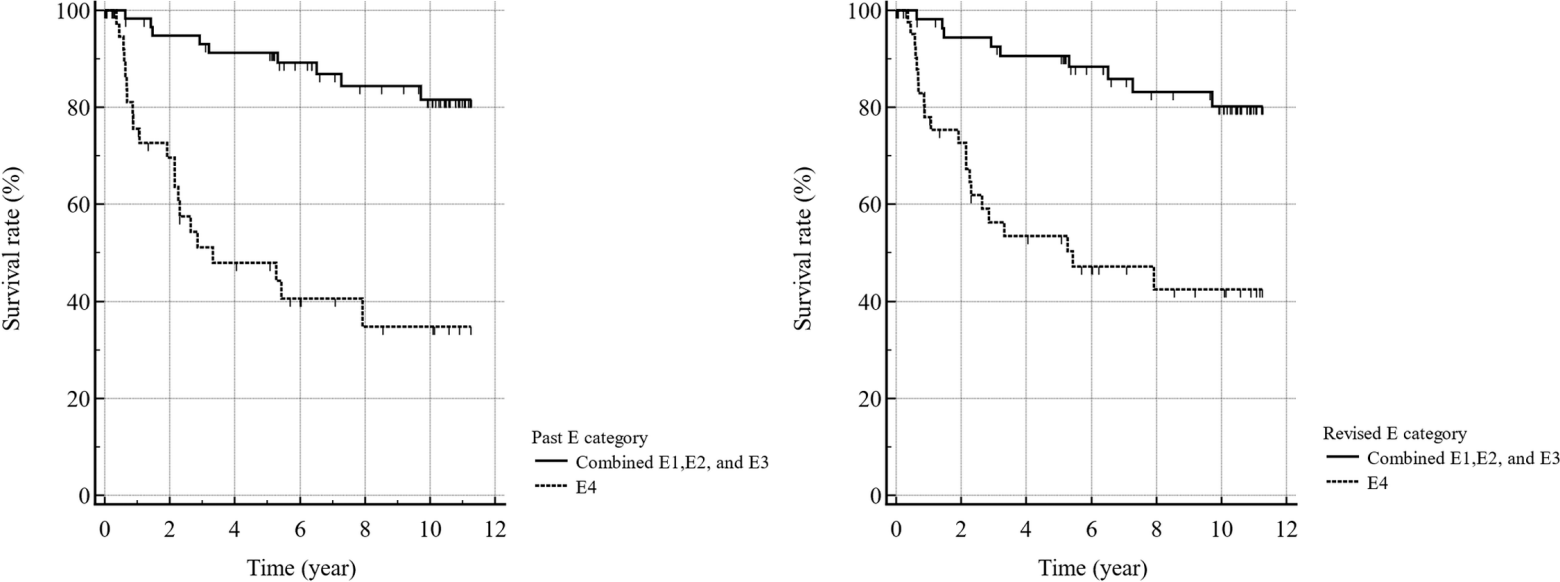
Kaplan–Meier survival curves for the Past (left) and Revised (right) E1, E2, and E3 categories vs. the E4 category. The Kaplan**–**Meier analysis comparing the combined E1, E2, and E3 categories with the E4 category revealed significant differences in the survival rates in the Past and Revised categories (*p* < 0.0001, Log-rank test). The 5-year survival rates were 91% for past E1, E2, and E3 and 48% for past E4, compared with 90% for revised E1, E2, and E3 and 53% for revised E4.

The mean OP was 1,338 days for patients with Past E4 findings (n = 40) in terms of prognosis post-CTC, in contrast to 2,719 days for patients with combined Past E1, E2, and E3 results (n = 63). The results of Kaplan–Meier analysis revealed that patients in the Past E4 category (mean survival: 5.6 years) had a significantly worse prognosis (*p* < 0.0001, log-rank test) compared to those with other extracolonic findings (mean survival: 10.1 years) across the remaining categories (E1, E2, and E3 combined) (Fig 1). The hazard ratio was found to be 8.2 (95%CI: 3.7–18.4) for the Past E1, E2, and E3 categories combined compared with the Past E4 category. Within the Past E4 category, no significant difference in survival was observed between groups in which CTC was performed for the primary complaint of colorectal cancer (mean survival: 8.7 years) and groups with primary complaints other than colorectal cancer (mean survival: 8.0 years). The Revised E category also showed significant differences in prognoses between the E1, E2, and E3 categories (mean survival: 10.0 years) and the E4 category (mean survival:6.2) (p < 0.0001) (Fig 2). The hazard ratio was 5.2 (95%CI: 2.4–11.2) for the Revised E1, E2, and E3 combined categories compared with the Revised E4 category.

## Discussion

Although CTC is expected to be an objective and standardized modality for both screening and secondary examinations, a potential concern regarding its integration into colorectal screening programs is the uncertainty surrounding the medium- and long-term outcomes of the advantages and potential risks associated with detecting extracolonic lesions. CTC inherently includes extracolonic organs from the abdomen to the pelvic region in its imaging range, and incidental findings such as lung, renal, and malignant lymphomas have been reported in low-dose screening CTC [7]. Colorectal cancer screening is primarily aimed at the early detection of colorectal cancer and the reduction of mortality through timely treatment in asymptomatic, average-risk individuals. Therefore, CTC screening includes potential negative aspects such as increased costs, physical and psychological burden, and time consumption caused by detecting extracolonic lesions that do not improve prognosis [8].

Screening CTC is performed using low-dose radiation, and preoperative CTC employs normal-dose imaging to provide a detailed evaluation of parenchymal organs. Contrast-enhanced CTC used in preoperative diagnosis provides important information regarding vascular anatomy and metastasis, as well as colorectal data, in a single examination [9]. Although gadolinium ethoxybenzyl-diethylenetriaminepentaacetic acid (EOB-DTPA) MRI is superior to contrast-enhanced CT for detecting liver metastases [10], contrast-enhanced CT remains essential for preoperative screening. In this study, many patients had underlying malignancies, including lymph node metastases (regional, intermediate, and distant) from various primary organs, as well as other suspicious malignant lesions. These findings were classified as the E4 category according to the C-RADS.

In our study, 10% of patients with Past E2 findings and 83% of those with Past E3 findings required follow-up. These follow-ups often necessitated additional examinations to rule out metastasis or other diseases. Follow-up for Past E4 findings frequently provided crucial information for clinical staging, affecting treatment plans in 78% of these cases. According to a previous report, potentially significant extracolonic findings on CTC were identified in 2.8% of asymptomatic patients and 5.2% of symptomatic patients [11]. In contrast, our study found that 39% of Past E4 patients and 45% of Revised E4 patients had potentially significant extracolonic findings, which is a considerably higher rate than those of other populations. Even in the general population, there is a possibility of detecting non-colorectal, poor-prognosis diseases classified as E4 findings.

Attending physicians, who carefully balance the potential benefits and risks of each additional examination, typically ordered follow-up clinical examinations. However, screening exams are usually uniformly conducted across many patients. One study proposed that the benefits of CTC outweigh its disadvantages with radiation exposure [12], whereas the drawbacks of identifying extracolonic lesions during CTC screenings can be difficult to assess or quantify. Category E3 findings are ambiguously defined as a “likely unimportant, incompletely characterized, and subject to local practice and patient preference” posing a significant challenge. The distinction between E3 and E4 becomes blurred in low-dose, non-contrast screening CTC, particularly in patients with unknown clinical histories. However, our study suggests that managing E4 findings, which are significant extracolonic lesions affecting prognosis, can provide essential guidance for clinicians in treatment planning.

The importance of analyzing both the mid-term and long-term benefits and drawbacks of E3, along with the prognostic implications of E4, is likely to increase in future colorectal CT screenings. Photon-counting CT, a less invasive imaging technology, may offer improved detection capabilities with lower radiation doses [13]. Additionally, machine learning holds promise for enhancing data processing by automating the analysis of large volumes of CT image data. However, further validation is required to fully assess the impact of these technologies on screening accuracy and the evaluation of extracolonic findings, particularly for E3 lesions. Future strategies will require more stratified data collection, as current screening practices predominantly focus on disease-specific mortality rates. This approach will involve comprehensive stratification, potentially utilizing machine learning, but further validation is needed to ensure the effective integration of the C-RADS or similar systems into data management based on patient background information to enhance the screening process.

CTC, like barium enema, provides an objective assessment of the colon, with the additional advantage of evaluating extracolonic lesions. In the present study, 18% of patients were diagnosed with gastric cancer and 6% with gynecological conditions, identified through the detection of seeding and adhesions. Previous studies have highlighted the utility of CTC in diagnosing gastric cancer seeding [14]. For precise diagnosis using CTC, a comprehensive approach is necessary, considering not only alterations in the colon wall but also extracolonic lesions. This broader diagnostic framework differs from the more focused approach needed for detecting colorectal polyps.

The introduction of the Past E and Revised E categories was intended to evaluate the evolution of diagnostic interpretations over time, as well as their consistency and reliability. Revised E involved a consensus reading by multiple radiologists, aiming to reduce individual biases and enhance the reliability of the diagnostic data. Among the cases initially classified as Past E1, E2, and E3, several were reclassified as Revised E4, showing regional lymph node metastasis (N1 or higher in the TNM classification) due to small clusters of lymph nodes observed near the lesion. Additionally, minor peritoneal dissemination or transperitoneal extension was more thoroughly evaluated, resulting in an upgrade to E4. One case of renal cell carcinoma misclassified as Past E2 was recategorized as Revised E4. This tumor, which was identified during follow-up after CTC and treated surgically, was pathologically diagnosed as pT1. In the Past E evaluation, the survival curves for E1 and E3 appeared to be inverted, suggesting unexpected outcomes. However, in the Revised E evaluation, these curves appeared to align more intuitively, reflecting a correction or adjustment. This observation suggests that the reclassification in Revised E improved the consistency and reliability of the survival rate comparison. The re-evaluation probably reduced the variability in categorization, leading to a more coherent differentiation between categories, which better reflects the true clinical prognosis.

This study was limited by its retrospective design and relatively small cohort. Efforts were made to reduce bias using continuous data and obtaining informed consent during the study period. The data sets and image viewers were not the most current, but their historical nature was crucial for observing mid-to-long-term outcomes. Two-dimensional imaging, which is predominantly used for extraintestinal lesion detection, has not seen substantial technological improvements because of the data collection period, despite significant advancements in three-dimensional image processing. The dated nature of the data and equipment is unlikely to have affected the study’s reliability or applicability. Most cases involved preoperative screening, with imaging conditions tailored to each patient’s situation, including dose and contrast media. It is important to recognize the potential of additional examinations needed to influence treatment pathways, particularly in patients who have achieved complete surgical resection of extracolonic lesions or a partial response to chemotherapy. However, the emergence of selection bias when these examinations are necessary complicates the rigorous assessment of their effect on treatment outcomes and prognosis. Another limitation of our study is that the final classification of the Past E category was determined by a single radiologist. While the past E-assessment reflected routine practice, the revised E-assessment was conducted under optimised conditions, including consensus among multiple radiologists, to increase reliability and consistency. By incorporating both the past E classification and the revised E classification, this study aimed to provide a balanced view from actual clinical data and an optimised, reliable assessment framework.

## Conclusion

Post-detection follow-up of extracolonic lesions in patients with E2 and E3 findings primarily involved exclusionary diagnoses without substantive treatment plan changes. The prognosis was worse for the patients with category E4 findings than for those with category E1, E2, and E3 findings among the extracolonic lesions detected during clinical CTC. Irrespective of the underlying disease, the E4 category finding is crucial in clinical CTC because it provides important guidance for clinicians and assists in the development of appropriate treatment plans.

## Supporting information

S1 Fig Chief complaint of patients for CTC. (DOCX)

S1 Table Numbers of lesions in each E category. (DOCX)

S2 Table Extracolonic lesions subjected to additional examination. (DOCX)

S3 Table Kaplan–Meier survival analysis. (XLSX)

S4 Table The original data for analysis. (XLSX)
