# Correction: Prognosis of extracolonic findings on clinical computed tomographic colonography: A single-center experience

**DOI:** 10.1371/journal.pone.0334534

**Published:** 2025-10-14

**Authors:** Hideto Tomimatsu, Keita Fujimoto, Taketo Suto, Masayuki Matsuo

In the Abstract, there is an error in the sixth sentence. The correct sentence is: Kaplan–Meier analysis revealed worse prognosis for E4 (mean survival: 5.6 years) than for E1–E3 combined (10.1 years) (p < 0.0001).

In the Results section, there is an error in the sixth sentence of the third paragraph. The correct sentence is: None of the patients in the Past E2 or E3 categories required further examinations that necessitated changes to the treatment plan (mean OP for E2: 2,880 days; mean OP for E3: 2,976 days).

In the Results, there is an error in the third and seventh sentence of the fourth paragraph. The correct third sentence is: The hazard ratio for Past E4 (vs the combined Past E1–E3) was 8.2 (95% CI: 3.7–18.4). The correct seventh sentence is: The hazard ratio for Revised E4 (vs the combined Revised E1–E3) was 5.2 (95% CI: 2.4–11.2).
