# Supplementary material for: Prognosis of extracolonic findings on clinical computed tomographic colonography: A single-center experience
Source: PLoS One. 2025 Feb 28;20(2):e0315601. doi: 10.1371/journal.pone.0315601 (PMC11870342; doi:10.1371/journal.pone.0315601)
Supplement: S1 Fig — (DOCX) [file pone.0315601.s001.docx]

Supporting information 1

Figure: Chief complaint of patients for CT colonography
