# Supplementary material for: Prognosis of extracolonic findings on clinical computed tomographic colonography: A single-center experience
Source: PLoS One. 2025 Feb 28;20(2):e0315601. doi: 10.1371/journal.pone.0315601 (PMC11870342; doi:10.1371/journal.pone.0315601)
Supplement: S1 Table — (DOCX) [file pone.0315601.s002.docx]

Supporting information 2.

Table: Numbers of lesion in each Revised E category

| CATEGORY | NUMBER OF PATIENTS | EXTRACOLONIC FINDINGS | NUMBER OF LESIONS  （INCLUDED DUPULICATE CASES） |
| --- | --- | --- | --- |
| Revised E1 | 7 |  | 0 |
| Revied E2 | 44 | Liver cyst  Fatty liver  Liver cavernous hemangioma  Cholecystolithiasis  Renal cyst  Renal Stone  Benign prostatic hypertrophy  Hernia  Myoma uteri  Other | 17  3  3  5  27  4  2  3  6  13 |
| Revised E3 | 6 | Lymph node adenopathy  Ovarian lesion  Pancreatic cyst  Liver cirrhosis  Common iliac aneurysm | 1  1  1  1  1 |
| Revised E4 | 46 | Liver metastasis  Lung metastasis  Lymph node metastasis  Peritoneal dissemination  Gastric tumor  Ovarian tumor  Renal or Renal pelvic tumor  Retroperitoneal tumor  Appendix tumor  Aortic aneurysm | 12  2  18  12  2  2  2  1  1  1 |
| TOTAL | 103 |  | 141 |

Table: Numbers of lesion in each Past E category

| CATEGORY | NUMBER OF PATIENTS | EXTRACOLONIC FINDINGS | NUMBER OF LESIONS  （INCLUDED DUPULICATE CASES） |
| --- | --- | --- | --- |
| Past E1 | 18 |  | 0 |
| Past E2 | 39 | Liver cyst  Fatty liver  Liver cavernous hemangioma  Cholecystolithiasis  Renal cyst  Renal Stone  Myoma uteri  Other | 11  3  3  3  16  2  5  3 |
| Past E3 | 6 | Lymph node adenopathy  Pancreatic cyst  Liver cirrhosis  Polycystic kidney | 2  2  1  1 |
| Past E4 | 40 | Gastric cancer  Liver metastasis  Lung metastasis  Lymph node metastasis  Peritoneal dissemination  Renal tumor  Ovarian tumor  Appendiceal tumor  Pelvic mass  Retroperitoneal tumor  Abdominal aortic aneurysm  Adhesion (cause of stenosis) | 3  7  1  16  7  2  1  1  2  1  1  1 |
| TOTAL | 103 |  | 95 |
