# Supplementary material for: Prognosis of extracolonic findings on clinical computed tomographic colonography: A single-center experience
Source: PLoS One. 2025 Feb 28;20(2):e0315601. doi: 10.1371/journal.pone.0315601 (PMC11870342; doi:10.1371/journal.pone.0315601)
Supplement: S2 Table — (DOCX) [file pone.0315601.s003.docx]

Supporting information 3.
Table: Extracolonic lesions subjected to additional examinations

| CATEGORY | AGE | SEX | MAIN COMPLAINT | EXTRACOLOLNIC　LESION | ADDITIONAL　EXAMINATION | RESULT OF ADDITIONAL　EXAMINATION | PLAN OR OUTCOME　OF TREATMENT |
| --- | --- | --- | --- | --- | --- | --- | --- |
| E2 | 60's | M | Sigmoid colon cancer | Prostatic hyperplasia | MRI | Benign Prostatic hyperplasia | Observation |
|  | 70's | M | Rectal cancer | Liver nodule | MRI | Cavernous hemangioma | Observation |
|  | 60's | M | Sigmoid colon cancer | Lung nodule | CT | No change in size | Observation |
|  | 70's | M | Transverse colon cancer,  Post gastrectomy | Lung nodule | CT | No change | Observation |
| E3 | 60's | M | Rectal cancer | Lung nodule (Ground glass nodule) | CT | Slightly enlarge | Under observation |
|  | 50's | F | Left ovarian cancer | Lung nodule | CT | No change | Observation |
|  | 60's | M | Gastric cancer | Multiple renal cysts | CT | Polycystic kidney | Consult to urology |
|  | 50's | M | Sigmoid colon cancer | Lung nodule | CT | No change | Observation |
|  | 70's | F | Sigmoid colon cancer | Lung nodule | CT | No change | Observation |
| E4 | 60's | M | Sigmoid colon cancer | Renal tumor,  Multiple lymph node swelling | PET-CT,  Biopsy | Squamous cell carcinoma | Chemotherapy |
|  | 80's | M | Ascending colon cancer | Aortic aneurysm | Enhanced CT | Aortic abdominal aneurysm (incidental finding) | Died of other illnesses |
|  | 70's | M | Sigmoid colon cancer | Liver nodule,  Lymph node swelling | MRI | Liver metastasis | Hepatectomy,  Chemotherapy |
|  | 60's | M | Rectal cancer | Renal pelvic tumor | Cytology | Renal pelvic cancer | Nephrectomy |
|  | 40's | M | Ascending colon cancer | Intra-pelvic tumor | PET-CT | Possibly Desmoid tumor (incidental finding) | Died of other illnesses |
|  | 70's | F | Sigmoid colon cancer | Multiple liver metastases  Lymph node metastases | MRI | Liver metastases | Chemotherapy |
|  | 60's | M | Esophageal cancer | Paraaortic lymph node swelling | PET-CT | Lymph node metastases | Chemotherapy |
|  | 60's | M | Transverse colon cancer | Liver metastasis | MRI | Liver metastasis | Hepatectomy |
|  | 60's | F | Rectal cancer | Multiple lung metastases,  Lymph node swelling | CT | Multiple lung metastases | Chemotherapy |
